# Catalogue of Rose Gall, Herb Gall, and Inquiline Gall Wasps (Hymenoptera: Cynipidae) of the United States, Canada and Mexico

**DOI:** 10.3897/BDJ.9.e68558

**Published:** 2021-08-24

**Authors:** Louis F. Nastasi, Andrew R. Deans

**Affiliations:** 1 Frost Entomological Museum, Penn State University, University Park, United States of America Frost Entomological Museum, Penn State University University Park United States of America

**Keywords:** biodiversity, checklist, Aulacideini, Ceroptresini, Diastrophini, Diplolepidini, Phanacidini, Synergini

## Abstract

**Background:**

Cynipidae (Hymenoptera: Cynipoidea) is a diverse group of wasps, many of which are capable of inducing plants to make galls, novel structures that protect and nourish the wasps' larvae. Other cynipids, especially those species in Ceroptresini and Synergini, are understood to be usurpers of galls made by other cynipids. The North American cynipid fauna has not been fully catalogued since 1979, but there is renewed interest in revising the taxonomy and in doing research that sheds light on the mechanisms of gall induction, the evolution of this life history, and their ecological interactions more broadly. Significant taxonomic changes have impacted the group since 1979, thereby warranting a new catalogue.

**New information:**

The current state of knowledge of species classified in Aulacideini, Ceroptresini, Diastrophini, Diplolepidini, Phanacidini and Synergini in the United States, Canada, and Mexico is summarised in catalogue format. We report 323 names, including 170 valid species of rose gall wasps, herb gall wasps, and inquiline gall wasps, classified in 12 genera, from the United States, Canada, and Mexico. Current taxonomic status, distribution, host associations, and vernacular names are listed for each species. The catalogue also includes the original description of galls for many species of gall-inducer, as well as atomised characterisations of different gall traits as key-value pairs. For most galling species without existing vernacular names, new vernacular names are proposed.

## Introduction

**Figure 1.**
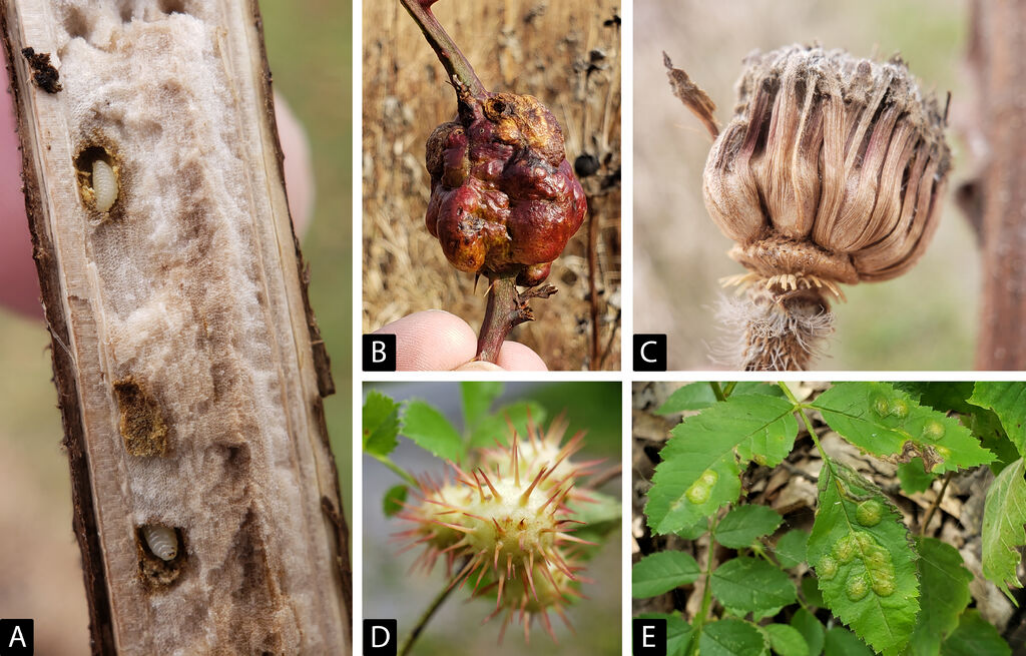
Examples of galls induced by some of the species treated in this catalogue. Photos are not at the same scale. **A.** *Antistrophusjeanae* Tooker & Hanks, 2004 inside *Silphiumperfoliatum* L. stems; **B.** *Diatrophusnebulosus* (Osten Sacken, 1861) on *Rubus* sp. stem; **C.** *Antistrophuslaciniatus* Gillette, 1891 galls on disc flowers of *Silphiumlaciniatum* L.; **D.** *Diplolepispolita* (Ashmead, 1890) galls on leaves of *Rosa* sp.; **E.** *Diplolepisrosaefolii* (Cockerell, 1889) galls in leaves of *Rosa* sp. Photos by Andrew R. Deans

The hymenopteran family Cynipidae constitutes a diverse assemblage of gall-forming and inquilinous wasps, which occur worldwide. While the galls induced by these insects are often conspicuous and charismatic (see Fig. 1), the North American fauna lacks robust classification and accessible diagnostic tools. The group has received moderate phylogenetic attention in recent years, but revisionary taxonomy remains uneven. While the higher taxonomy of the family is uncertain, species that induce galls or act as inquilines are well-distinguished from other Cynipoidea, both in terms of their current taxonomy and natural history.

The present work catalogues the species of tribes Aulacideini, Ceroptresini, Diastrophini, Diplolepidini, Phanacidini and Synergini that are known from Canada, the United States, and Mexico. The higher taxonomy at the tribe level employed herein follows that of Buffington et al. (2020) and Ronquist et al. (2015). Cynipini, which is by far the most species-rich of the family's tribes, is excluded from the present work, primarily due to the existence of many taxonomic works in press or in preparation. While Cynipini is the most diverse of the family's tribes, the herb gallers (Aulacideini, Diastrophini, and Phanacidini), rose gallers (Diplolepidini), and inquilines (Ceroptresini and Synergini) comprise a significant portion of the family's diversity and, therefore, are worthy of their own treatment.

The most recent previous catalogues of the family are Weld (1957), Weld (1959), Weld (1960), Burks (1979), and a database contributed to the Hymenoptera Online database by Johan Liljeblad (Johnson et al. 2019), but many changes to the taxonomy of Cynipidae warrant the development of a new catalogue to reflect recent improved knowledge.

## Materials and methods

Data were compiled using the Darwin Core data standard (Wieczorek et al. 2012) and the Darwin Core Extensions for taxon description, species distribution, and vernacular names. An initial list of cynipid species was obtained by examining the Cynipidae dataset in the Hymenoptera Online database (Johnson et al. 2019). Existing literature relating to the Cynipidae of North America was reviewed in order to generate records relating to the taxonomy, distribution, and biology of cynipid wasps. Data from the community science platform iNaturalist (2021) were also used in order to substantiate locality records and provide the most complete possible distribution for the covered species. iNaturalist observations used in this manner were personally examined by the authors and have been verified to a reasonable degree of certainty to properly represent the organisms contained in the records. Vernacular names were assigned for gall-inducing species that have not yet been assigned one.

Gall descriptions from the literature are provided verbatim for many species. These descriptions were also atomised into individual traits and presented as key-value pairs, using a controlled vocabulary for gall terminology (Deans et al. 2021; included also in the Darwin Core Archive version of this data set). Host associations for numerous Synergini are provided verbatim from the given references (see Suppl. material 1). All association data were indexed by the Global Biotic Interactions resource (GloBI; Poelen et al. 2014).

## Data resources

The catalogue of Aulacideini, Ceroptresini, Diastrophini, Diplolepidini, Phanacidini, and Synergini of the United States, Canada, and Mexico is provided as Suppl. material 1 (Darwin Core Archive) and provides 322 species-level and subspecific names including synonyms. We recognise 170 valid species. The taxonomy of many of these species needs revision, and the intention is to issue subsequent versions of this catalogue to reflect substantial changes as they are made in the future. A checklist, which summarises the data set in the Darwin Core Archive, including nomenclature, notes on biology and geographic distribution, is provided below.

Initial data were drawn from the Hymenoptera Online Database (Norm Johnson; Johan Liljeblad). We also used occurrence data from iNaturalist, when we could confidently verify the species' identity, to further establish or even expand the distributions for many species. Botanical taxonomic names were matched to names in the Taxonomic Name Resolution Service.

## Checklists

### The Aulacideini, Ceroptresini, Diastrophini, Diplolepidini, Phanacidini, and Synergini of the United States, Canada, and Mexico

#### Cynipidae

C9E23CEA-1FD2-50C2-8FA9-BB8C14AF514E

#### Aulacideini

E838A317-9E8F-5254-9680-5D5E3D9A5450

#### Antistrophus

Walsh, 1869

549B226B-14FF-5C97-A4F2-0F82059845CD

#### Antistrophus bicolor

Gillette, 1891

3CC98A0A-2E09-5414-BF11-D2808804EBE4

##### Ecological interactions

###### Feeds on

May induce galls on *Silphiumintegrifolium* Michx., but claim is unsubstantiated

##### Distribution

United States: Illinois

#### Antistrophus chrysothamni

(Beutenmüller, 1908)

83DE067C-5FF6-521F-AD32-FEEA3EF400E3

Aulax chrysothamni Beutenmüller, 1908

##### Ecological interactions

###### Feeds on

Induces galls on *Chrysothamnus* Nutt.

##### Distribution

United States: Arizona

#### Antistrophus jeanae

Tooker & Hanks, 2004

CA8554B7-3ED5-505C-86A2-D504C1391D15

##### Ecological interactions

###### Feeds on

Induces galls on *Silphiumperfoliatum* L.

##### Distribution

United States: Illinois

#### Antistrophus laciniatus

Gillette, 1891

975091D4-2C70-5FE1-A7F8-8D6C9EA96AA8

##### Ecological interactions

###### Feeds on

Induces galls on *Silphiumlaciniatum* L.

##### Distribution

United States: Illinois, Kansas, Texas, Wisconsin

#### Antistrophus lygodesmiaepisum

Walsh, 1869

6A58B073-A013-581C-8A37-FB63159A2E55

Antistrophus pisum Ashmead, 1885 | *Ascepiadiphilastephanotidis* Ashmead, 1897

##### Ecological interactions

###### Feeds on

Induces galls on *Lygodesmiajuncea* D. Don ex Hook

##### Distribution

United States: Colorado, Iowa, Kansas, Missouri, Nebraska, Oregon, South Dakota, Texas; Canada: Alberta, Saskatchewan

#### Antistrophus meganae

Tooker & Hanks, 2004

DAEE0DAA-09CA-54F4-ACDA-7B2E57489387

##### Ecological interactions

###### Feeds on

Induces galls on *Silphiumterebinthinaceum* Jacq.

##### Distribution

United States: Illinois

#### Antistrophus microseris

(McCracken & Egbert, 1922)

C53547A9-43C4-5485-B1CB-75B2B2742478

Aylax microseris McCracken & Egbert, 1922

##### Ecological interactions

###### Feeds on

Induces galls on *Microseris* D. Don

##### Distribution

United States: California

#### Antistrophus minor

Gillette, 1891

41688252-03C3-5567-B0A0-A273AD17DA43

Aulax Kieffer, 1902

##### Ecological interactions

###### Feeds on

Induces galls on *Silphiumlaciniatum* L. and possibly other *Silphium*

##### Distribution

United States: Illinois

#### Antistrophus rufus

Gillette, 1891

8C7634D3-1487-5C2D-8428-94C71ECA9F4A

##### Ecological interactions

###### Feeds on

Induces galls on *Silphiumlaciniatum* L.

##### Distribution

United States: Illinois, Kansas

#### Antistrophus silphii

Gillette, 1891

BFDB85D3-AC55-56B0-9C04-681B17FD70BD

Antistrophus leavenworthi Bassett, 1900

##### Ecological interactions

###### Feeds on

Induces galls on *Silphiumintegrifolium* Michx. and *S.perfoliatum* L.

##### Distribution

United States: Georgia, Illinois, Indiana, Iowa, Kansas, Missouri, Nebraska, Virginia, Wisconsin

#### Aulacidea

Ashmead, 1897

9C12FA9B-1048-5647-B4C0-AFBCFD6B4679

#### Aulacidea abdita

Kinsey, 1920

815B184A-18F8-53B3-8016-8B74D5754E9C

##### Ecological interactions

###### Feeds on

Induces galls on *Lactucacanadensis* L. and possibly additional *Lactuca* L. species

##### Distribution

Canada: Québec

#### Aulacidea acroptilonica

Tyurebaev, 1972

3CF43C1D-0C84-55D5-A82D-84F88BC59FD2

##### Ecological interactions

###### Feeds on

Induces galls on *Acroptilonrepens* (L.) DC.

##### Distribution

United States: Colorado, Montana, Wyoming

#### Aulacidea ambrosiaecola

(Ashmead, 1896)

936A5A46-5733-5FA7-BA40-E9F79F6C35F1

Aulax ambrosiaecola Ashmead, 1896

##### Ecological interactions

###### Feeds on

May induce galls on *Ambrosia* L. sp., but claim is unverified

##### Distribution

United States: Missouri

#### Aulacidea annulata

Kinsey, 1920

123E873C-0775-5ECE-AF7B-68F026738C76

##### Ecological interactions

###### Feeds on

Induces galls on *Lactuca* L. sp. or possibly Prenanthes

##### Distribution

United States: Massachusetts, Nebraska

#### Aulacidea harringtoni

(Ashmead, 1887)

A137D651-08C4-5FD0-AC53-BD6F21F3B53F

Aulax harringtoni Ashmead, 1887 | *Aulaxbicolor* Gillette, 1891 | *Aulaxmulgediicola* Ashmead, 1896 | *Aulaxcavicola* Ashmead, 1896

##### Ecological interactions

###### Feeds on

Induces galls on *Lactucafloridana* L.

##### Distribution

United States: Connecticut, District of Columbia, Illinois, Massachusetts, Missouri, North Carolina, New Jersey, New York, Oklahoma; Canada: Ontario

#### Aulacidea nabali

(Brodie, 1892)

79CC50ED-00FF-5DA0-B2D8-7EACB3912CDF

Aulax nabali Brodie, 1892

##### Ecological interactions

###### Feeds on

Induces galls on *Nabalusaltissimus* (L.) Hook

##### Distribution

United States: Massachusetts, New Jersey, New York; Canada: Ontario

#### Aulacidea pilosellae

(Kieffer, 1901)

E8A225A7-9DFD-57BC-B8FE-365174B5B995

Aulax beijerinckii Kieffer, 1899 | *Aulaxpilosellae* Kieffer, 1901

##### Ecological interactions

###### Feeds on

Induces galls on *Pilosella* Hill spp.

##### Distribution

United States (Possibly introduced)

#### Aulacidea podagrae

(Bassett, 1890)

DC384C6F-37A9-58B7-9993-121571B1E926

Aulax podagrae Bassett, 1890

##### Ecological interactions

###### Feeds on

Induces galls on *Lactucacanadensis* L.

##### Distribution

United States: District of Columbia, Illinois, Kansas, New York, Ohio, Pennsylvania, Virginia

#### Aulacidea subterminalis

Niblett, 1946

0952212D-9DB9-5B7B-B667-55AA45701DD8

##### Ecological interactions

###### Feeds on

Induces galls on *Hieracium* L. spp.

##### Distribution

United States (Possibly introduced); Canada: British Columbia (Introduced)

#### Aulacidea tumida

(Bassett, 1890)

2BF6844F-A8B4-59BF-A21A-728CDA042FB6

Aulax tumidus Bassett, 1890 | *Aulaxsonchicola* Ashmead, 1896 | *Aulacideasolidaginis* Girault, 1903

##### Ecological interactions

###### Feeds on

Induces galls on *Lactucacanadensis* L.

##### Distribution

United States: Massachusetts, New Hampshire, Virginia

#### Liposthenes

Förster, 1869

69297998-B43F-5B0A-A4D6-513A48B7538D

#### Liposthenes glechomae

(Linnaeus, 1758)

D7BABC3C-4B74-5F20-9102-936B059D8DF3

Cynips Linnaeus, 1758 | *Diastrophussimilis* Bassett, 1881 | *Aulax Latreillei* Kieffer, 1898

##### Ecological interactions

###### Feeds on

Induces galls on *Glechomahederacea* L.

##### Distribution

United States: Connecticut, Delaware, Iowa, Illinois, Indiana, Kansas, Massachusetts, Maryland, Michigan, Minnesota, Nebraska, New Jersey, New York, Ohio, Pennsylvania, Rhode Island, Vermont

#### Ceroptresini

F6177428-2A02-5654-99BA-D162D623C5FD

#### Buffingtonella

Lobato-Vila & Pujade-Villar, 2019

8224F093-FB3A-5D53-A853-9C341E94BAB5

#### Buffingtonella politus

(Ashmead, 1896)

269CA989-78E5-5926-B363-0C28CB438087

Ceroptres politus Ashmead, 1896

##### Ecological interactions

###### Feeds on

Host unknown but presumed to be inquilinous

##### Distribution

United States: Virginia

#### Ceroptres

Hartig, 1840

73439C7C-793D-5AAD-863F-38463F8B1D90

#### Ceroptres catesbaei

Ashmead, 1885

E5F637CD-9FB0-5D07-9037-6A16CB84F0CE

##### Ecological interactions

###### Feeds on

Inquiline of: galls of *Callirhytisquercuscatesbaei* Ashmead, 1881 on *Quercuslaevis* Walter

##### Distribution

United States: Florida

#### Ceroptres confertus

(McCracken & Egbert, 1922)

0B7356DB-0CA3-5653-8B1F-33C1C8D8BAF3

Periclistus confertus McCracken & Egbert, 1922

##### Ecological interactions

###### Feeds on

Inquiline of: galls of *Andricusconfertus* McCracken & Egbert, 1922 on *Quercuslobata* Née

##### Distribution

United States: California

#### Ceroptres cornigera

Melika & Buss, 2002

68DADFAA-E49E-5229-BEDF-43CDA7DFB8BB

##### Ecological interactions

###### Feeds on

Inquiline of: galls of *Callirhytisquercuscornigera* (Osten Sacken 1865) on *Quercuspalustris* Münchh.

##### Distribution

United States: Kentucky

#### Ceroptres ensiger

(Walsh, 1864)

EFF06F76-BD1E-55E8-A5AC-0D7046BA16C6

Amblynotus ensiger Walsh, 1864

##### Ecological interactions

###### Feeds on

Inquiline of: galls of *Andricusquercuspetiolicola* (Bassett, 1863) on *Quercusbicolor* Willd.

##### Distribution

United States: Illinois

#### Ceroptres frondosae

Ashmead, 1896

DD1530AA-2F28-5E80-A03C-26970BC42666

##### Ecological interactions

###### Feeds on

Inquiline of *Andricusquercusfrondosus* (Bassett, 1865) [as Cynips?quercusfrondosa] on *Quercusprinoides* Willd.

##### Distribution

United States: Missouri

#### Ceroptres junquerasi

Lobato-Vila & Pujade-Villar, 2019

34739E6F-7422-5A72-977F-9360E16B2CFD

##### Ecological interactions

###### Feeds on

Inquiline of: galls of *Drosperlentum* Kinsey, 1937 on *Quercusobtusata* Humb. and Bonpl. and *Quercuslaeta* Liebm.; galls of *Andricussphaericus* Pujade-Villar, 2016 on *Quercusrugosa* Née and *Quercuslaeta* Liebm.; galls of *Andricusgeorgei* Pujade-Villar, 2011 on *Quercuscandicans* Née; galls of *Neuroteruseugeros* Pujade-Villar, 2018 on *Quercuslaeta* Liebm.; galls of *Atruscapictor* (Kinsey, 1936) on *Quercus* sp.; galls of *Atrusca* sp. on *Quercusobtusata* Humb. and Bonpl.; unknown galls on *Quercusacutifolia* Née; unknown galls on *Quercuslaeta* Liebm.; galls of *Melikaiellaamphibolensis* Pujade-Villar, 2014 on *Quercuscastanea* Née; unknown leaf galls of *Neuroterus* on *Quercuslaeta* Liebm.

##### Distribution

Mexico: Ciudad de México, Jalisco, México, Michoacán de Ocampo, Morelos, Puebla

#### Ceroptres lanigerae

Ashmead, 1885

084D7283-9F17-5FCD-A921-D149F578024E

##### Ecological interactions

###### Feeds on

Inquiline of: galls of *Andricusquercuslanigera* (Ashmead, 1881) on *Quercusminima* (Sarg.) Small, *Quercusoleoides* Schltdl. & Cham., and *Quercusvirginiana* Mill. (= *Q.geminata* Small)

##### Distribution

United States: Florida

#### Ceroptres lenis

Lobato-Vila & Pujade-Villar, 2019

5B0CBA21-69FB-5155-BE54-A5146FE40735

##### Ecological interactions

###### Feeds on

Inquiline of: unknown galls on *Quercuslaeta* Liebm.

##### Distribution

Mexico: Ciudad de México

#### Ceroptres mexicanus

Lobato-Vila & Pujade-Villar, 2019

08C7A3C4-69D0-552A-BEF1-FEF42EB77710

##### Ecological interactions

###### Feeds on

Inquiline of: unknown spherical leaf galls on *Quercus* sp.

##### Distribution

Mexico: México

#### Ceroptres minutissimi

Ashmead, 1885

61A47EBB-195C-5225-B67B-6D3DC531AB4E

##### Ecological interactions

###### Feeds on

Inquiline of: galls of *Neuroterusquercusminutissimus* (Ashmead, 1885) on *Quercusvirginiana* Mill.; undetermined, non-deciduous galls on leaves of *Quercusrugosa* Née

##### Distribution

United States: Florida; Mexico: Ciudad de México

#### Ceroptres montensis

Weld, 1957

46755285-1D45-52D8-A8CF-AE68B60FDA3D

##### Ecological interactions

###### Feeds on

Inquiline of: galls of *Andricusreniformis* McCracken & Egbert, 1922 on *Quercusvaccinifolia* Kell.; galls of *Disholcaspistruckeensis* (Ashmead, 1896) on *Quercuschrysolepis* Liebm.

##### Distribution

United States: California, Oregon

#### Ceroptres nigricus

Lobato-Vila & Pujade-Villar, 2019

BFEE20F8-69F8-50F1-AECA-D1F554F86CAB

##### Ecological interactions

###### Feeds on

Inquiline of: undetermined galls on *Quercusacutifolia* Née; undetermined twig galls on *Quercus* sp.

##### Distribution

Mexico: México, Michoacán de Ocampo

#### Ceroptres petiolicola

(Osten Sacken, 1861)

4A2E7CFF-02E1-526A-A766-03B79405AD79

Amblynotus ? petiolicola Osten Sacken, 1861

##### Ecological interactions

###### Feeds on

Inquiline of: galls of *Andricusquercuspetiolicola* (Bassett, 1863) on *Quercusmichauxii* Nuttall (= *Q.prinusauctores* not L., = Q.prinusvar.michauxii (Nutt.) Chapman 1860); galls of *Neuroterusquercusbatatus* (Fitch, 1859) [unverified]

##### Distribution

United States: District of Columbia; Canada: Québec

#### Ceroptres pisum

(Osten Sacken, 1861)

BE03673F-2E35-5E0B-94E8-F17D76E2BC6A

Sarothrus ? pisum Osten Sacken, 1861

##### Ecological interactions

###### Feeds on

Inquiline of: galls of *Acraspispezomachoides* (Osten Sacken, 1862) on *Quercusalba* L. [unverified]

##### Distribution

United States: District of Columbia

#### Ceroptres quadratifacies

Lobato-Vila & Pujade-Villar, 2019

ED19BC4F-8651-5C58-870E-8EF496207477

##### Ecological interactions

###### Feeds on

Inquiline of: galls of *Atrusca* sp. on *Quercusmicrophylla* Née; galls of Sphaeroterasnr.pulchripennis on *Quercusmicrophylla* Née

##### Distribution

Mexico: Aguascalientes

#### Ceroptres quercusobtusilobae

(Karsch, 1880)

8F31A8D6-A067-5616-94B5-7A1A8BBF59D8

Diplolepis q. obtusilobae Karsch, 1880 | *Cynipsobtusilobae* (Karsch, 1880) | *Neuroterusquercusobtusilobae* (Karsch, 1880) | *Ceroptresobtusilobensis* (Karsch, 1880)

##### Ecological interactions

###### Feeds on

Inquiline of: undetermined woody, tuberous galls on *Quercusstellata* Wengenh. (= *Q.obtusiloba* Michx.)

##### Distribution

United States: Texas

#### Ceroptres rufiventris

Ashmead, 1896

D378947A-D671-54DF-8B79-42F5A3B950E2

##### Ecological interactions

###### Feeds on

Inquiline of: galls of *Amphibolipsquercusostensackenii* (Bassett, 1863) on *Quercuscoccinea* Müenchh. and *Quercuspalustris* Müenchh.

##### Distribution

United States: Missouri

#### Ceroptres snellingi

Lyon, 1996

3D7D9563-DD5B-5F85-85B7-C3C2CE90F4A5

Ceropteres [sic] *snellingi* Lyon, 1996

##### Ecological interactions

###### Feeds on

Inquiline of: galls of *Andricusflocculentus* Lyon, 1996 on *Quercuspungens* Liebm.; galls of *Andricussphaericus* Pujade-Villar, 2016 on *Quercusmexicana* Bonpl.; galls of *Andricussphaericus* Pujade-Villar, 2016 on *Quercusrugosa* Née; galls of *Andricus* sp. on leaves of Quercuscfchihuahuensis Trel.; galls of *Andricus* sp. on leaves of Quercuscfeduardi Trel.; undetermined leaf petiole galls on *Q.potosina* Trel.; galls of *Andricus* sp. (*georgei* group) on *Quercus* sp.

##### Distribution

United States: Texas; Mexico: Aguascalientes, Ciudad de México, México, Nuevo León, Zacatecas

#### Diastrophini

09FA6A09-4321-546D-9B4E-AB209BDBFF0B

#### Diastrophus

Hartig, 1840

D1314A1B-4BDA-53F7-99C4-C4D436D8FFC2

#### Diastrophus austrior

Kinsey, 1922

9ECD1868-4EB9-5CF3-9F60-A32E4961B895

Diastrophus kincaidii var. austrior Kinsey, 1922

##### Ecological interactions

###### Feeds on

Induces galls on *Rubusparviflorus* Nutt. and *R.nutkanus* Moc. ex Ser.

##### Distribution

United States: California

#### Diastrophus bassettii

Beutenmüller, 1892

9D592593-819A-5140-A041-D4461363EAD9

##### Ecological interactions

###### Feeds on

Induces galls on *Rubusprocumbens* Muhl. and *R.hispidus* L.

##### Distribution

United States: Connecticut, Massachusetts, North Carolina, New Jersey, New York, Rhode Island

#### Diastrophus cuscutaeformis

Osten Sacken, 1863

4994CBFB-3355-5BBB-896A-DD30A0688FFE

##### Ecological interactions

###### Feeds on

Induces galls on *Rubus* L. spp.

##### Distribution

United States: Illinois, Indiana, Massachusetts, Maryland, Maine, Michigan, Minnesota, Missouri, North Carolina, New Hampshire, New Jersey, New York, Ohio, Pennsylvania, South Carolina, Tennessee, Virginia, Vermont; Canada: Newfoundland and Labrador, Ontario

#### Diastrophus fragariae

Beutenmüller, 1915

2642D025-2851-5CF7-8CBA-E8B640FFFFC6

##### Ecological interactions

###### Feeds on

Induces galls on *Fragariavirginiana* Duch.

##### Distribution

United States: Michigan, Minnesota; Canada: Ontario

#### Diastrophus fusiformans

Ashmead, 1890

D29E704F-3060-57E7-BB24-19A76E9C3E9D

##### Ecological interactions

###### Feeds on

Induces galls on *Potentilla* L. sp.

##### Distribution

United States: Colorado, Washington

#### Diastrophus kincaidii

Gillette, 1893

46C6D395-9504-5F48-80E3-949F4AAADC43

Diastrophus minimus Bassett, 1900

##### Ecological interactions

###### Feeds on

Induces galls on *Rubusnutkanus* Moc. ex Ser. and *R.parviflorus* Nutt.

##### Distribution

United States: California, Idaho, Michigan, Minnesota, Montana, Oregon, Washington, Wisconsin; Canada: British Columbia, Ontario

#### Diastrophus nebulosus

(Osten Sacken, 1861)

897B39C5-E276-5E15-A157-348160A122D6

Cynips (Diastrophus?) nebulosus Osten Sacken, 1861

##### Ecological interactions

###### Feeds on

Induces galls on *Rubuscorchorifolius* L. f. (=*villosus*)

##### Distribution

United States: Arkansas, Connecticut, District of Columbia, Delaware, Georgia, Illinois, Indiana, Kentucky, Louisiana, Massachusetts, Maryland, Michigan, Missouri, Mississippi, North Carolina, New Jersey, New York, Ohio, Oklahoma, Pennsylvania, South Carolina, Tennessee, Texas, Virginia, Vermont, West Virginia; Canada: Ontario

#### Diastrophus niger

Bassett, 1900

3130653A-F6C1-54E7-8DFD-54C08D38517B

##### Ecological interactions

###### Feeds on

Induces galls on *Potentillacanadensis* L.

##### Distribution

United States: Connecticut, Massachusetts, Minnesota, New Jersey, New York, Rhode Island

#### Diastrophus piceus

Provancher, 1886

C7F825A9-55A3-59E5-B99C-E95F4AED6537

##### Ecological interactions

###### Feeds on

Gall unknown

##### Distribution

Canada: Ontario

#### Diastrophus potentillae

Bassett, 1864

CCA85FB4-E004-538E-BC09-B6273C5D7544

Gonaspis potentillae (Bassett, 1864)

##### Ecological interactions

###### Feeds on

Induces galls on *Potentillacanadensis* L. and *P.simplex* Michx.

##### Distribution

United States: Illinois, Indiana, Massachusetts, Maine, Minnesota, North Carolina, Ohio; Canada: New Brunswick, Nova Scotia

#### Diastrophus radicum

Bassett, 1870

A4D0054E-FE4A-547B-8ABA-69D8289B5045

##### Ecological interactions

###### Feeds on

Induces galls on *Rubuscorchorifolius* L. f. (= *villosus*) and *R.occidentalis* L.

##### Distribution

United States: Colorado, Michigan, North Carolina

#### Diastrophus smilacis

Ashmead, 1896

F1A1DC6B-52F5-502F-9283-66F8B53C455D

##### Ecological interactions

###### Feeds on

Host unknown; previously suspected to induce galls on *Smilaxrotundifolia* L. and *S.herbacea* L., but this claim has been refuted (Gates et al. 2020)

##### Distribution

United States: Florida, Illinois

#### Diastrophus tumefactus

Kinsey, 1920

8A54AAD9-7674-54F1-9079-742E618A74F9

##### Ecological interactions

###### Feeds on

Induces galls on *Tridophyllumnorvegicum* (L.) Greene (=Potentillamonspeliensisvar.norvegica)

##### Distribution

Canada: Ontario, Québec

#### Diastrophus turgidus

Bassett, 1870

63B39EC1-C452-5F4C-8CF4-B8C7B1D4164F

Diastrophus turdigus [sic] Bassett, 1870

##### Ecological interactions

###### Feeds on

Induces galls on *Rubusstrigosus* Michx.

##### Distribution

Canada: Manitoba, Newfoundland and Labrador, Ontario, Québec; United States: Colorado, Maine, Minnesota, New York, Ohio, Vermont

#### Periclistus

Förster, 1869

84E1BC3E-04FD-598C-A1C9-A2565DE7F2BB

#### Periclistus arefactus

McCracken & Egbert, 1922

BC0151EC-E347-53EE-953B-563A8B6CBD63

##### Ecological interactions

###### Feeds on

Inquiline of: galls of *Diplolepisarefacta* (Gillette, 1894) on *Rosacalifornica*

##### Distribution

United States: California

#### Periclistus californicus

Ashmead, 1896

8EE72E7E-FC2B-5705-A2D9-B31663DD81F6

##### Ecological interactions

###### Feeds on

Inquiline of: galls of *Diplolepispolita* (Ashmead, 1890)

##### Distribution

United States: California, Colorado, Wyoming

#### Periclistus obliquus

Provancher, 1888

0F5452DA-073D-5FCD-909F-83F11C8A2E83

##### Ecological interactions

###### Feeds on

Host unknown but presumed to be inquilinous

##### Distribution

United States: California

#### Periclistus piceus

Fullaway, 1911

1CD8FB37-F8F9-57C8-9E44-7D96F5A68323

##### Ecological interactions

###### Feeds on

Inquiline of: galls of *Diplolepispolita* (Ashmead, 1890) on *Rosacalifornica*

##### Distribution

United States: California; Canada: Alberta, Ontario

#### Periclistus pirata

(Osten Sacken, 1863)

FEA9087C-8BA6-595A-80D0-CB141D79DADA

Aulax pirata Osten Sacken, 1863 | *Rhoditesglobulus* Beutenmüller, 1892

##### Ecological interactions

###### Feeds on

Inquiline of: galls of *Diplolepisignota* (Osten Sacken, 1863)

##### Distribution

United States: District of Columbia; Canada: Alberta, Ontario, Saskatchewan

#### Periclistus semipiceus

(Harris, 1841)

43C06708-5A9B-5CC3-8F99-762D348D4054

Cynips semipiceus Harris, 1841

##### Ecological interactions

###### Feeds on

Inquiline of: undetermined subspherical galls on roots of *Rosa* sp.

##### Distribution

United States: Massachusetts

#### Periclistus smilacis

Ashmead, 1896

24730EDD-A238-5CA5-8685-2632AFF373D7

##### Ecological interactions

###### Feeds on

Inquiline of: galls of *Diastrophussmilacis* Ashmead, 1896 [unverified]

##### Distribution

United States: Florida

#### Synophromorpha

Ashmead, 1903

A2B35999-0824-550A-A052-DC7E6C7ECEE5

#### Synophromorpha kaulbarsi

Ritchie & Shorthouse, 1987

E09640CC-69AC-5265-A3AC-F14F8CF38913

##### Ecological interactions

###### Feeds on

Host unknown but presumed to be inquilinous

##### Distribution

Mexico: Puebla

#### Synophromorpha rubi

Weld, 1952

1D2BB20E-AB7A-5A50-A79E-140D72903EF1

##### Ecological interactions

###### Feeds on

Inquiline of: galls of *Diastrophuscuscutaeformis* Osten Sacken, 1863 on *Rubus* spp.

##### Distribution

United States: Illinois, Massachusetts, Ohio, Rhode Island; Canada: Ontario

#### Synophromorpha sylvestris

(Osten Sacken, 1861)

27A8F6BC-F2AB-5D62-8685-C2EF702B4D09

Synophrus sylvestris Osten Sacken, 1861

##### Ecological interactions

###### Feeds on

Inquiline of: galls of *Diastrophusnebulosus* (Osten Sacken, 1861); galls of *Diastrophusturgidus* Bassett, 1870; galls of *Diastrophusbassettii* Beutenmüller, 1892 on *Rubus* spp.

##### Distribution

United States: Arkansas, Connecticut, District of Columbia, Florida, Illinois, Indiana, Massachusetts, Maryland, Maine, Missouri, North Carolina, New Jersey, New York, Ohio, Oklahoma, Pennsylvania, Rhode Island, Tennessee, Virginia, West Virginia

#### Synophromorpha terricola

Weld, 1952

D2DC8ECC-A765-54F1-A7C3-D36E3B208A38

##### Ecological interactions

###### Feeds on

Inquiline of: galls of *Diastrophusbassettii* Beutenmüller, 1892; galls of *Diastrophuscuscutaeformis* Osten Sacken, 1863; galls of *Diastrophusradicum* Bassett, 1870 on *Rubus* spp.

##### Distribution

United States: Alabama, Connecticut, Maryland, North Carolina, New York, Ohio, Virginia, West Virginia

#### Diplolepidini

0B6CF8AA-30F5-50E6-9647-7AC0F40BFAF6

#### Diplolepis

Geoffroy, 1762

1509E157-CC25-58D8-9629-3FFA41B35CFF

#### Diplolepis arefacta

(Gillette, 1894)

BEB59E19-C7BE-5CDB-940B-DB45158247AB

Rhodites arefactus Gillette, 1894

##### Ecological interactions

###### Feeds on

Induces galls on *Rosa* L. sp.

##### Distribution

United States: Colorado, Utah

#### Diplolepis ashmeadi

(Beutenmüller, 1918)

D413FC25-851C-5F5B-A268-2B8E46A1F835

Rhodites ashmeadi Beutenmüller, 1918

##### Ecological interactions

###### Feeds on

Induces galls on *Rosanutkana* C. Presl

##### Distribution

United States: Oregon

#### Diplolepis bassetti bassetti

(Beutenmüller, 1918)

039BF13C-D8EF-5024-8798-AD0F1AD99323

Rhodites bassetti Beutenmüller, 1918

##### Ecological interactions

###### Feeds on

Induces galls on *Rosanutkana* C. Presl and *R.woodsii* Lindl.

##### Distribution

United States: Oregon; Canada: Alberta, British Columbia, Saskatchewan

#### Diplolepis bassetti lucida

Kinsey, 1922

15278AAE-6FC6-5224-B446-46CBD25F1560

Diplolepis bassetti var. lucida Kinsey, 1922

##### Ecological interactions

###### Feeds on

Induces galls on *Rosanutkana* C. Presl

##### Distribution

United States: Idaho, Oregon

#### Diplolepis bicolor

(Harris, 1841)

52BD0394-A25B-59C0-8C3C-AA8B2720D9CD

Cynips bicolor Harris, 1841

##### Ecological interactions

###### Feeds on

Induces galls on *Rosawoodsii* Lindl. and *R.arkansana* Porter

##### Distribution

United States: Arkansas, California, Connecticut, Idaho, Illinois, Indiana, Kentucky, Maryland, Maine, Michigan, Minnesota, Missouri, Montana, North Carolina, New Hampshire, New Jersey, New York, Ohio, Pennsylvania, Washington, Wisconsin; Canada: Alberta, British Columbia, Manitoba, New Brunswick, Nova Scota, Ontario, Québec, Saskatchewan

#### Diplolepis californica

(Beutenmüller, 1914)

B7F78F6C-72EB-540D-82A6-DDA6086F1482

Rhodites californicus Beutenmüller, 1914

##### Ecological interactions

###### Feeds on

Induces galls on *Rosa* L. sp.

##### Distribution

United States: California, Idaho

#### Diplolepis dichlocera

(Harris, 1841)

F4665822-C060-55C1-A268-4A5EF7E9C3FF

Cynips dichlocera Harris, 1841

##### Ecological interactions

###### Feeds on

Induces galls on *Rosa* L. sp.

##### Distribution

United States: Massachusetts; Canada: Ontario

#### Diplolepis fulgens

(Gillette, 1894)

429F31D8-C402-53D0-94E5-BF758634E95B

Rhodites fulgens Gillette, 1894

##### Ecological interactions

###### Feeds on

Induces galls on *Rosarugosa* Thunb.

##### Distribution

United States: Illinois, New York, South Dakota

#### Diplolepis fusiformans fusiformans

(Ashmead, 1890)

3D783558-D47B-507E-8FE3-DA2B6C4C5AC8

Rhodites fusiformans Ashmead, 1890

##### Ecological interactions

###### Feeds on

Induces galls on *Rosaarkansana* Porter, *R.woodsii* Lindl., and *R.blanda* Aiton

##### Distribution

United States: Arizona, Colorado, Idaho, Illinois, Minnesota, Nebraska; Canada: Alberta, British Columbia, Ontario

#### Diplolepis fusiformans mendocinensis

Kinsey, 1922

00C37F3F-3AF1-5289-BA5F-82F9161D5BFD

Diplolepis fusiformans var. mendocinensis Kinsey, 1922

##### Ecological interactions

###### Feeds on

Induces galls on *Rosa* L. sp.

##### Distribution

United States: California

#### Diplolepis fusiformans minuta

Kinsey, 1922

06D7ED59-2313-505D-AC41-62D3567C9ABC

Diplolepis fusiformans var. minuta Kinsey, 1922

##### Ecological interactions

###### Feeds on

Induces galls on *Rosa* L. sp.

##### Distribution

United States: California

#### Diplolepis gracilis

(Ashmead, 1897)

53CABA93-598E-5516-939E-08B2587A29EB

Rhodites graxilis Ashmead, 1897

##### Ecological interactions

###### Feeds on

Induces galls on *Rosa* L. sp.

##### Distribution

United States: Minnesota, Wisconsin; Canada: Alberta, British Columbia, Saskatchewan

#### Diplolepis ignota

(Osten Sacken, 1863)

71C93824-F4EA-5B48-B058-6A7C7F850DD6

Rhodites ignota Osten Sacken, 1863 | *Rhoditescarolina* Ashmead, 1887

##### Ecological interactions

###### Feeds on

Induces galls on *Rosaarkansana* Porter, *R.blanda* Aiton, *R.carolina* L., *R.nitida* Willd., and *R.virginiana* Mill.

##### Distribution

United States: Colorado, Connecticut, District of Columbia, Iowa, Illinois, Indiana, Massachusetts, Montana, North Carolina, New Jersey, New York, Pennsylvania, Washington; Canada: Manitoba, Saskatchewan

#### Diplolepis inconspicuis

Dailey & Campbell, 1973

C33D529F-41E8-5BE5-BEDC-881D6ED67C37

##### Ecological interactions

###### Feeds on

Induces galls on *Rosacalifornica* Cham. and Schltdl.

##### Distribution

United States: California

#### Diplolepis lens

Weld, 1952

AD9DA108-52AF-5E7E-85B4-9FE0BD712CCB

##### Ecological interactions

###### Feeds on

Induces galls on *Rosanutkana* C. Presl

##### Distribution

United States: California, Oregon, Washington

#### Diplolepis mayri

(Schlechtendal, 1877)

FC548EC7-A329-5740-83F7-2D1A8EDA2827

Rhodites mayri Schlechtendal, 1877

##### Ecological interactions

###### Feeds on

Induces galls on *Rosarubiginosa* L.

##### Distribution

United States: New Jersey

#### Diplolepis nebulosa

(Bassett, 1890)

E0A744AB-8C02-59C0-9AAD-3E19C42E58BA

Rhodites nebulosa Bassett, 1890

##### Ecological interactions

###### Feeds on

Induces galls on *Rosablanda* Aiton, *R.carolina* L., *R.rubiginosa* L., and *R.woodsii* Lindl.

##### Distribution

United States: Connecticut, New York; Canada: Alberta, Ontario

#### Diplolepis neglecta

(Gillette, 1894)

9086F863-9539-5161-A63F-1FC48A084602

Rhodites neglecta Gillette, 1894

##### Ecological interactions

###### Feeds on

Induces galls on *Rosa* L. sp.

##### Distribution

United States: Colorado

#### Diplolepis nervosa

(Curtis, 1838)

4FEC4B9C-596F-5E61-8DF9-2360738D15C0

Diplolepis centifoliae (Hartig, 1840) | *Rhoditescentifoliae* Hartig, 1840 | *Rhoditesandrei* Kieffer, 1904 | *Rhoditesdispar* Niblett, 1943 | *Rhoditeskiefferi* Loiselle, 1912 | *Diplolepisrosarum* (Giraud, 1859) | *Rhoditesrosarum* Giraud, 1859 | *Cynipsnervosa* Curtis, 1838 |

##### Ecological interactions

###### Feeds on

Induces galls on *Rosacanina* L. and *R.rubiginosa* L.

##### Distribution

United States: Oregon; Canada: Ontario, Québec

#### Diplolepis nodulosa

(Beutenmüller, 1909)

EB9DD194-383A-5322-B9EE-6F8D95B4F74A

Rhodites nodulosus Beutenmüller, 1918

##### Ecological interactions

###### Feeds on

Induces galls on *Rosawoodsii* Lindl., Rosavirginiana Mill., and *R.blanda* Aiton; possibly induces galls onRosacarolinavar.lucida (Ehrh.) Farw. [unsubstantiated]

##### Distribution

United States: Illinois, Massachusetts; Canada: Alberta, British Columbia, Ontario, Prince Edward Island, Saskatchewan

#### Diplolepis oregonensis

(Beutenmüller, 1918)

25D0813E-DC76-5B63-B05D-6AEC276E3567

Rhodites oregonensis Beutenmüller, 1918

##### Ecological interactions

###### Feeds on

Induces galls on *Rosanutkana* C. Presl

##### Distribution

United States: Idaho, Oregon, Washington; Canada: Saskatchewan

#### Diplolepis ostensackeni

(Beutenmüller, 1918)

1482054B-ED1A-5B4C-A9CB-186335291A87

Rhodites ostensackeni Beutenmüller, 1918

##### Ecological interactions

###### Feeds on

Induces galls on *Rosanutkana* C. Presl

##### Distribution

United States: Oregon

#### Diplolepis polita

(Ashmead, 1890)

CC2EF917-439A-55FC-BB66-555CCEB63D13

Rhodites polita Ashmead, 1890 | *Rhoditesoccidentalis* Beutenmüller, 1922

##### Ecological interactions

###### Feeds on

Induces galls on *Rosacalifornica* Cham. & Schltdl., *R.woodsii* Lindl., *R.acicularis* Lindl., and *R.arkansana* Porter

##### Distribution

United States: Alaska, California, Colorado; Canada: Alberta, British Columbia, Manitoba, Ontario, Québec, Saskatchewan, Yukon

#### Diplolepis pustulatoides

(Beutenmüller, 1914)

234F952F-46D7-5B93-8A44-6A2EB7184DC4

Rhodites pustulatoides Beutenmüller, 1914

##### Ecological interactions

###### Feeds on

Induces galls on *Rosa* L. sp.

##### Distribution

United States: Indiana

#### Diplolepis radicum

(Osten Sacken, 1863)

D8396AD6-A863-58BF-B8C8-427CE0A292BB

Rhodites radicum Osten Sacken, 1863 | *Tribaliabatatorum* Walsh, 1864 | *Rhoditesatahensis* Bassett, 1890 | Diplolepisradicumvar.johnsoni Kinsey, 1922 | Diplolepisradicumvar.plana Kinsey, 1922 | Diplolepisradicumvar.divergens Kinsey, 1922 | Diplolepisradicumvar.angustior Hunter, 1923

##### Ecological interactions

###### Feeds on

Produces galls on *Rosacarolina* L., *R.nutkana* C. Presl, and *R.woodsii* Lindl.

##### Distribution

United States: Colorado, Connecticut, District of Columbia, Illinois, Indiana, Massachusetts, Minnesota, North Carolina, New Jersey, New York, Ohio, Oregon, Pennsylvania, Washington; Canada: British Columbia, Manitoba, Ontario

#### Diplolepis rosae

(Linnaeus, 1758)

22EB17CE-6F45-5466-8BDB-449DEB99FB91

Cynips rosae Linnaeus, 1758

##### Ecological interactions

###### Feeds on

Induces galls on *Rosarugosa* Thunb., *R.cinnamoea* L., *R.rubiginosa* L., and *R.blanda* Aiton

##### Distribution

United States: California, Colorado, District of Columbia, Idaho, Illinois, Kansas, Massachusetts, Maine, Michigan, Montana, North Carolina, New Hampshire, New York, Ohio, Oregon, Pennsylvania, Rhode Island, South Carolina, Utah, Virginia, Vermont, Washington, West Virginia; Canada: Alberta, British Columbia, New Brunswick, Newfoundland and Labrador, Nova Scotia, Ontario, Québec

#### Diplolepis rosaefolii

(Cockerell, 1889)

6D6E9B48-D25F-5F78-A084-294071807CEB

Rhodites rosaefolii Cockerell, 1889 | *Rhoditeslenticularis* Bassett,

##### Ecological interactions

###### Feeds on

Induces galls on *Rosaacicularis* Lindl., *R.woodsii* Lindl., and *R.arkansana* Porter

##### Distribution

United States: California, Colorado, Illinois, Pennsylvania, Washington; Canada: Alberta, British Columbia, Manitoba, Newfoundland and Labrador, Nova Scotia, Ontario, Saskatchewan, Yukon

#### Diplolepis similis

(Ashmead, 1896)

BAE12AAA-1448-5592-A968-788EAA299EC7

Rhodites similis Ashmead, 1897

##### Ecological interactions

###### Feeds on

Induces galls on *Rosa* L. sp.

##### Distribution

United States: Wyoming

#### Diplolepis spinosa

(Ashmead, 1887)

F1E70807-DF28-5599-A4C8-485924EFA8FE

Rhodites spinosa Ashmead, 1887 | *Rhoditesspinosissima* Gillette, 1889 | *Rhoditesmultispinosa* Gillette, 1890

##### Ecological interactions

###### Feeds on

Induces galls on *Rosarubiginosa* L., *R.woodsii* Lindl., and *R.blanda* Aiton

##### Distribution

United States: California, Colorado, Florida, Idaho, Illinois, Massachusetts, Maine, Minnesota, Washington; Canada: Alberta, British Columbia, Manitoba, Ontario, Saskatchewan

#### Diplolepis terrigena

Weld, 1952

3ECE4BEC-32BB-5716-9E10-1E2EB524B0AC

##### Ecological interactions

###### Feeds on

Induces galls on *Rosa* L. sp.

##### Distribution

United States: California

#### Diplolepis triforma

Shorthouse & Ritchie, 1984

6233ED3E-1DC4-520A-9EE1-FF8F530646A2

##### Ecological interactions

###### Feeds on

Induces galls on *Rosaacicularis* Lindl. and *R.woodsii* Lindl.

##### Distribution

United States: Minnesota; Canada: Alberta, Manitoba, Ontario, Saskatchewan

#### Diplolepis tuberculator

(Cockerell, 1888)

DF5CAF41-2099-5204-9CD4-1EAA4F5B3266

Diplolepis tuberculatrix (Cockerell, 1888) | *Rhoditestuberculator* Cockerell, 1888 | Diplolepistuberculatrixvar.coloradensisformcoloradensis Kinsey & Ayres, 1922 | Diplolepistuberculatrixvar.coloradensisformsubcoloradensis Kinsey & Ayres, 1922 | Diplolepistuberculatrixvar.wasatchensis Kinsey & Ayres, 1922 | Diplolepistuberculatrixvar.versicolor Kinsey & Ayres, 1922 | Diplolepistuberculatrixvar.melanderi Kinsey & Ayres, 1922 | Diplolepistuberculatrixvar.rubriderma Kinsey & Ayres, 1922 | Diplolepistuberculatrixvar.sierranensis Kinsey & Ayres, 1922 | Diplolepistuberculatrixvar.descansonis Kinsey & Ayres, 1922

##### Ecological interactions

###### Feeds on

Induces galls on *Rosa* L. sp.

##### Distribution

United States: California, Colorado, North Dakota, New Mexico, Oregon, Utah, Washington

#### Diplolepis tuberculosa

(Osten Sacken, 1861)

717B9C2F-45F3-5C2F-9DC8-62EE4460C6BC

Cynips (Rhodites?) tuberculosa Osten Sacken, 1861

##### Ecological interactions

###### Feeds on

Induces galls on *Rosa* L. sp.

##### Distribution

United States

#### Diplolepis tumida

(Bassett, 1890)

AF93C998-465D-5713-AA9D-D70387A71921

Rhodites tumidus Bassett, 1890 | Diplolepistuberculatrixvar.tumidaformxerophila Kinsey and Ayres, 1922

##### Ecological interactions

###### Feeds on

Produces galls on *Rosa* L. sp.

##### Distribution

United States: Utah

#### Diplolepis variabilis

(Bassett, 1890)

8C002A32-DA40-5EDE-AD10-5E68FD2477F2

Rhodites variabilis Bassett, 1890 | *Rhoditesglobuloides* Beutenmüller, 1907

##### Ecological interactions

###### Feeds on

Induces galls on *Rosawoodsii* Lindl.

##### Distribution

United States: Colorado, Itado, Texas, Utah, Washington, Wyoming; Canada: British Columbia

#### Diplolepis verna

(Osten Sacken, 1863)

73EEC23D-093B-514A-8AB8-99A48C4A1F3D

Rhodites verna Osten Sacken, 1863

##### Ecological interactions

###### Feeds on

Induces galls on *Rosablanda* Aiton

##### Distribution

United States: District of Columbia, New York

#### Diplolepis weldi

(Beutenmüller, 1913)

1B3554DB-203B-54C7-BFE9-FAC521F01E9E

Rhodites weldi Beutenmüller, 1913

##### Ecological interactions

###### Feeds on

Induces galls on *Rosa* L. sp.

##### Distribution

United States: California

#### Phanacidini

B528C46E-A0F4-5772-A058-E1354129B0FE

#### Phanacis

Förster, 1860

477FC6F2-9889-5176-85B4-C3758B53E2CB

#### Phanacis hypochoeridis

(Kieffer, 1887)

A5B7D1F6-DF35-5EDB-AF12-ECEE96C90790

Aulax hypochaeridis Kieffer, 1887

##### Ecological interactions

###### Feeds on

Induces galls on *Hypochaerisradicata* L.

##### Distribution

United States: California, Oregon; Canada: British Columbia

#### Phanacis taraxaci

(Ashmead, 1897)

4B67B9EB-3E06-553E-9182-BDBD27926D33

Gillettea taraxaci Ashmead, 1897

##### Ecological interactions

###### Feeds on

Induces galls on *Taraxacumofficinale* F. H. Wigg.

##### Distribution

United States: Minnesota, Pennsylvania

#### Synergini

A60DA05C-81C8-5EA2-BF7D-AFE155A7D723

#### Saphonecrus

Dalla Torre & Kieffer, 1910

F924F48B-D261-51DE-AFDC-B7E15DFD9DCA

#### Saphonecrus favanus

Weld, 1944

DEB82DC5-C059-5458-964D-1E7F7C109148

##### Distribution

United States: District of Columbia, Missouri

#### Saphonecrus gemmariae

(Ashmead, 1885)

15554AB1-8B73-50FE-9D4B-638EE69552A5

##### Distribution

United States: Florida

#### Synergus

Hartig, 1840

EACD37F8-0892-5C1D-9062-2D2703B39827

#### Synergus agrifoliae

Ashmead, 1896

8EBF588B-BB68-5D27-8AC9-628423F2253D

Synergus maculatus Fullaway, 1911 | *Synergusobscurus* McCracken & Egbert, 1922

##### Ecological interactions

###### Feeds on

Inquiline of: galls of *Neuroterussaltatorius* (Edwards, 1874); undetermined round, brown galls, 2.0 mm in diameter located on adaxial leaf surface on *Quercusagrifolia* (Lobato-Vila and Pujade-Villar 2021); undetermined galls on *Quercusgarryana* Douglas ex Hook

##### Distribution

United States: California

#### Synergus ashmeadi

Lobato-Vila & Pujade-Villar, 2021

D0B74DCE-122C-586D-B1CB-08307C513EEF

##### Ecological interactions

###### Feeds on

Inquiline of: galls of *Andricus* sp. on *Quercuspotosina*

##### Distribution

Mexico: Aguascalientes

#### Synergus aurofacies

Lobato-Vila & Pujade-Villar, 2020

3FC6ABA0-51C6-55A3-8887-3DEC1C8140C7

##### Ecological interactions

###### Feeds on

Inquiline of: galls of *Femuroslusum* Kinsey, 1937

##### Distribution

Mexico: Ciudad de México

#### Synergus atra

Gillette, 1896

CBB3F732-41D8-5CA4-9BE4-62AA9B979821

##### Ecological interactions

###### Feeds on

Inquiline of: galls of *Disholcaspisrubens* (Gillette, 1893) (asexual generation)

##### Distribution

United States: Colorado, Michigan

#### Synergus atripennis

Ashmead, 1896

4D08CD13-722B-59E4-9CDD-F289244E11DD

##### Ecological interactions

###### Feeds on

Inquiline of: galls of *Disholcaspisspongiosa* (Karsch, 1880) (asexual generation)

##### Distribution

United States: Florida

#### Synergus atripes

Gillette, 1896

696E30E4-C313-5F8F-82E1-A7334A6D728C

##### Ecological interactions

###### Feeds on

Inquiline of: galls of *Atruscabrevipennata* (Gillette, 1893) (asexual generation)

##### Distribution

United States: Colorado

#### Synergus batatoides

Ashmead, 1885

FF0A40DB-9088-5E6E-BEEF-DFA04B16AC05

##### Ecological interactions

###### Feeds on

Inquiline of: galls of *Callirhytisquercusbatoides* (Ashmead, 1881) (asexual generation)

##### Distribution

United States: Florida

#### Synergus bellus

McCracken & Egbert, 1922

4E1C2A11-F37B-5FB3-9F1F-34B26DA5AF89

##### Ecological interactions

###### Feeds on

Inquiline of: undetermined galls on *Quercuschrysolepis* Liebm.

##### Distribution

United States: California

#### Synergus beutenmulleri

Lobato-Vila & Pujade-Villar, 2021

D9734D66-951D-5254-AC75-6C53E1E950E1

##### Ecological interactions

###### Feeds on

Inquiline of: galls of *Andricus* sp. *Q.potosina*

##### Distribution

Mexico: Aguascalientes

#### Synergus brevicornis

Ashmead, 1896

27EE075D-3813-5722-8816-0155B5FA18B4

Saphonecrus brevicornis (Ashmead, 1896)

##### Ecological interactions

###### Feeds on

Inquiline of: galls of *Andricus* sp. on *Quercuswislizeni* A. DC.

##### Distribution

United States: California

#### Synergus bicolor

Ashmead, 1885

145F452C-ABDD-5417-A5D4-F62E5AA8EA55

##### Ecological interactions

###### Feeds on

Inquiline of: galls of *Andricusquercusfoliatus* (Ashmead, 1881)

##### Distribution

United States: Florida

#### Synergus campanula

Osten Sacken, 1865

27286621-38F3-5397-AFC3-ECCAB920007C

##### Ecological interactions

###### Feeds on

Inquiline of: galls of *Andricusdimorphus* (Beutenmüller, 1913); galls of *Acraspiserinacei* (Beutenmüller, 1909); galls of *Acraspispezomachoides* (Osten Sacken, 1862); galls of *Disholcaspisquercusglobulus* (Fitch, 1859); galls of *Disholcaspisquercusmamma* (Walsh, 1869); galls of Trigonaspis?quercusforticorne (Walsh, 1864)

##### Distribution

United States: District of Columbia, Illinois, Iowa, Kansas, Kentucky, Missouri, New Jersey, New York, Wisconsin

#### Synergus castanopsidis

(Beutenmüller, 1918)

9FBE5B43-97BF-536F-AFF6-D5EDCA212A0B

Periclistus castanopsidis (Beutenmüller, 1918)

##### Ecological interactions

###### Feeds on

Inquiline of: galls of *Dryocosmuscastanopsidis* (Beutenmüller, 1917) (asexual generation)

##### Distribution

United States: California

#### Synergus cibriani

Pujade-Villar & Lobato-Vila, 2017

D2C35873-BE59-5AAE-9ACB-00626E6B05BE

##### Ecological interactions

###### Feeds on

Inquiline of: undetermined spherical galls, possibly induced by *Disholcaspis*, on twigs of undetermined species of *Quercus*; undetermined spherical galls on abaxial leaf surface on *Quercusglabrescens* (Lobato-Vila and Pujade-Villar 2021)

##### Distribution

Mexico: Michoacán de Ocampo

#### Synergus citriformis

(Ashmead, 1885)

AE9272BC-5B5D-504C-BBE1-B1FD383087EA

Ceroptres citriformis Ashmead, 1885 | *Synergusniger* Fullaway, 1911 | *Synerguselegans* Nieves-Aldrey & Medianero, 2011

##### Ecological interactions

###### Feeds on

Inquiline of: "... a gall related to *Amphibolipscitriformis* Ashmead" (Lobato-Vila and Pujade-Villar 2021); galls of *Amphibolips*, *Disholcaspis*, *Cynips*, *Andricus*, *Dros*, and *Antron* spp. on *Quercus* spp. in sections *Quercus* and *Lobatae* (Lobato-Vila and Pujade-Villar 2021).

##### Distribution

United States: California, Florida; Mexico: Aguascalientes, Ciudad de México, Guanajuato, Hidalgo, México, Michoacán de Ocampo, Morelos, Oaxaca, Puebla, Tlaxcala, Veracruz de Ignacio de la Llave, Zacatecas

#### Synergus compressus

Lobato-Vila & Pujade-Villar, 2021

8942BA2E-C3A2-5672-A73D-BA63D7C28080

##### Ecological interactions

###### Feeds on

Inquiline of: tuberous galls probably induced by *Andricus* on *Quercuscrassifolia*

##### Distribution

Mexico: Puebla

#### Synergus confertus

McCracken & Egbert, 1922

636096D7-0FE3-5801-8328-80A6C83FB6FF

##### Ecological interactions

###### Feeds on

Inquiline of: galls of *Andricusconfertus* McCracken & Egbert, 1922

##### Distribution

United States: California

#### Synergus coniferae

Ashmead, 1885

8042433C-8620-5F59-A7DA-6A5DEC6BAD92

##### Ecological interactions

###### Feeds on

Inquiline of: galls of *Callirhytisquercusventricosa* (Bassett, 1864)

##### Distribution

United States: Florida, Iowa, Missouri

#### Synergus digressus

McCracken & Egbert, 1922

EFDD1074-DBF8-5F20-AF2D-D279BB130298

##### Ecological interactions

###### Feeds on

Inquiline of: undetermined twig galls on *Quercusagrifolia* Née

##### Distribution

United States: California

#### Synergus dimorphus

Osten Sacken, 1865

D426DE5C-D906-5AE6-821B-C74AAC647D59

##### Ecological interactions

###### Feeds on

Inquiline of: "woody twig galls on red oaks" (Gillette 1896); galls of *Besbicusconspicuus* (Kinsey, 1930) on *Quercuslobata* (Lobato-Vila and Pujade-Villar 2021)

##### Distribution

United States: California, District of Columbia, Michigan

#### Synergus distinctus

McCracken & Egbert, 1922

53E50119-FB79-5D99-9653-0DF2530F93A3

##### Ecological interactions

###### Feeds on

Inquiline of: galls of *Disholcaspiscanescens* (Bassett, 1890)

##### Distribution

United States: California

#### Synergus diversicolor

Lobato-Vila & Pujade-Villar, 2021

1A1109BB-1320-5D85-98B1-A00A8938E014

##### Ecological interactions

###### Feeds on

Inquiline of: galls of *Melikaiellabicolor* Pujade-Villar, 2014; galls of *Sphaeroteras* sp. on the leaves of *Quercusobtusata*; galls of *Neuroterus* sp. on the leaves of *Quercuspeduncularis*; undetermined round galls on the twigs of *Quercusacutifolia*

##### Distribution

Mexico: Hidalgo, Jalisco, Morelos, Puebla

#### Synergus dorsalis

(Provancher, 1888)

71FF879C-2BE8-510F-B082-639B7201716F

Synergus splendidus Fullaway, 1911 | *Synergusdubiosus* Fullaway, 1911 | *Ceroptresdorsalis* Provancher, 1888

##### Ecological interactions

###### Feeds on

Inquiline of: undetermined galls on *Quercuslobata*; galls of *Amphibolipsquercuspomiformis* (Bassett 1881) on *Quercusagrifolia*; *Synergusdubiosus* was reared from galls of *A.quercuspomiformis* on *Q.agrifolia*; undetermined galls on *Quercuswislizeni*. See Lobato-Vila and Pujade-Villar (2021) for more details.

##### Distribution

United States: California

#### Synergus duricorius

Gillette, 1896

EB061D7E-7793-5860-BA92-50B533CD9F60

##### Ecological interactions

###### Feeds on

Inquiline of: galls of *Disholcaspisquercusmamma* (Walsh, 1869)

##### Distribution

United States: Delaware, Minnesota

#### Synergus ebenus

Lobato-Vila & Pujade-Villar, 2021

430FE4F8-2944-5FBE-92CD-FB36DA299EF0

##### Ecological interactions

###### Feeds on

Inquiline of: galls of *Femurosrepandae* Kinsey, 1937; undetermined galls of *Neuroterus* on the leaves of *Quercusobtusata*; undetermined galls of *Andricus* on the twigs of *Quercusobtusata*

##### Distribution

Mexico: Ciudad de México, México, Veracruz de Ignacio de la Llave

#### Synergus equihuai

Pujade-Villar & Lobato-Vila, 2016

FCF0B99E-6219-54E1-B32F-7ACB6C6E6427

##### Ecological interactions

###### Feeds on

Inquiline of: undetermined concealed galls in the acorns of *Quercusrugosa*

##### Distribution

Mexico: Morelos

#### Synergus erinacei

Gillette, 1896

9717D9FD-38C4-568F-BD1F-5F796A68D6CA

##### Ecological interactions

###### Feeds on

Inquiline of: galls of *Acraspiserinacei* (Beutenmüller, 1909); galls of *Acraspismacrocarpae* Bassett, 1890; galls of *Acraspispezomachoides* Osten Sacken, 1862

##### Distribution

United States: Iowa, Illinois, Kentucky, New York; Canada: Ontario

#### Synergus estradae

Pujade-Villar & Lobato-Vila, 2016

C049B6A0-F4DB-5256-AB7F-09A80B655743

##### Ecological interactions

###### Feeds on

Inquiline of: woody, tuberous galls induced by *Andricus* spp. (Lobato-Vila and Pujade-Villar 2021)

##### Distribution

Mexico: Hidalgo, Morelos, Puebla

#### Synergus ficigerae

Ashmead, 1885

86762878-1B32-5663-AFF1-CFC673771E31

##### Ecological interactions

###### Feeds on

Inquiline of: galls of *Disholcaspis* sp. on *Quercusmagnoliifolia*

##### Distribution

United States: Florida, Virginia

#### Synergus filicornis

Cameron, 1883

5F182503-1F1F-596F-A4E6-EC3319FB7060

Synergus similis Gillette, 1896 | *Synergusfurnessana* Weld, 1913

##### Ecological interactions

###### Feeds on

Inquiline of: woolly galls of *Andricusfurnessae* (Weld, 1913); woolly galls of *Andricusmexicanus* Kinsey (= *Andricusnievesaldreyi* Pujade-Villar, 2011); woolly galls of *Andricuscrystallinus*; woolly galls of *Andricusgeorgei*; woolly galls of *Andricusmaesi*; woolly galls of *Cynips* spp. (*Antron* group); woolly galls of *Andricusnievesaldreyi*; woolly galls of *Andricusquercuslanigera* (Ashmead) (= *A.linaria* (Kinsey)); non-woolly galls of *Andricusrochai* Pujade-Villar, 2018; non-woolly galls of *Atrusca* spp.; non-woolly galls of Disholcaspisnr.insulana; non-woolly galls of Disholcaspisnr.mexicana; non-woolly galls of Kokkocynipsnr.doctorrosae; non-woolly galls of *Loxaulus*; undetermined galls on Quercussect.Quercus. According to Lobato-Vila and Pujade-Villar (2021), *Synergusfilicornis* was described from specimens "obtained from woolly leaf galls of ‘*Andricusguatemalensis* (Cameron, 1883)’", a name they consider incertae sedis.

##### Distribution

Mexico: Aguascalientes, Ciudad de México, Guanajuato, Jalisco, México, Michoacán de Ocampo, Morelos, Puebla, Querétaro, Zacatecas

#### Synergus flavens

McCracken & Egbert, 1922

554448EA-1BE1-5EA2-A45A-37668AE151A4

Synergus variegatus McCracken & Egbert, 1922

##### Ecological interactions

###### Feeds on

Inquiline of: galls of *Heteroecusflavens* (McCracken & Egbert, 1922); galls of *Heteroecuspacificus* (Ashmead, 1896); galls of *Trichoterascoquilletti* Ashmead, 1897

##### Distribution

United States: California

#### Synergus forcadellae

Lobato-Vila & Pujade-Villar, 2020

FF846158-1C0B-5489-97AB-CF2E5B4FA6DA

##### Ecological interactions

###### Feeds on

Inquiline of: woolly galls of *Andricus* sp(p).; woolly galls of *Striatoandricus* sp(p).; non-woolly galls of *Andricus* spp.; non-woolly galls of *Atrusca* spp.; non-woolly galls of *Amphibolips* spp.; non-woolly galls of *Cynips* spp. mainly on Quercussect.Quercus (Lobato-Vila and Pujade-Villar 2021)

##### Distribution

Mexico: Ciudad de México, Guanajuato, México, Michoacán de Ocampo, Morelos, Querétaro

#### Synergus gilletti

Pujade-Villar & Lobato-Vila, 2017

613C55C6-4876-5803-93C6-01F3820C7644

##### Ecological interactions

###### Feeds on

Inquiline of: galls of *Atrusca* sp. on *Quercuslaeta* and *Quercusrugosa*; galls of *Andricus* on the leaves of *Quercuslaeta*

##### Distribution

Mexico: Zacatecas

#### Synergus grahami

Lobato-Vila & Pujade-Villar, 2019

9B7A72B8-3169-58B1-A8C1-C2B34A84F899

##### Ecological interactions

###### Feeds on

Inquiline of: galls of *Andricussantafe* Pujade-Villar, 2013; undetermined galls, possibly *Andricus* or *Loxaulus*, on *Quercuscrassipes* and *Quercuslaeta*; galls of *Loxauluslaeta* Pujade-Villar, 2014

##### Distribution

Mexico: Ciudad de México

#### Synergus incisus

Gillette, 1896

823A9887-63D5-59D2-98FA-4582AE0C9EE4

##### Ecological interactions

###### Feeds on

Inquiline of: galls of *Callirhytisfrequens* (Gillette, 1892)

##### Distribution

United States: Colorado, New Mexico

#### Synergus laeviventris

(Osten Sacken, 1861)

68CD31AD-70FB-55F6-8AA2-63E09BAE7083

##### Ecological interactions

###### Feeds on

Inquiline of: galls of *Amphibolipscookii* Gillette, 1888; galls of *Amphibolipsconfluenta* (Harris, 1841); galls of *Amphibolipsquercusinanis* Osten Sacken, 1862; galls of *Amphibolipsquercusjuglans* (Osten Sacken, 1862); galls of *Amphibolipsquercusostensackenii* (Bassett, 1863); galls of *Amphibolipsquercusspongifica* (Osten-Sacken, 1862); galls of *Andricuspisiformis* Beutenmüller, 1911; galls of *Andricusquercusfrondosus* (Bassett, 1865); galls of *Atruscaquercuscentricola* (Osten-Sacken, 1861); galls of *Disholcaspisquercusglobulus* (Fitch, 1839); galls of *Disholcaspisrubens* (Gillette, 1893); undetermined gall on *Quercusfalcata* Michx.; undetermined bud gall on undetermined Quercussect.Lobatae

##### Distribution

United States: Colorado, District of Columbia, Illinois, Iowa, Michigan, Missouri, Ohio, Pennsylvania

#### Synergus lignicola

(Osten Sacken, 1862)

B826BAF5-DA46-51B4-AA2C-D882FC54D6F5

Cynips (*Synerges* [sic]) *lignicola* Osten Sacken, 1862 | *Synergusdavisi* (Beutenmüller, 1907) | *Andricusdavisi* Beutenmüller, 1907 | *Synerges* [sic] *rhoditiformis* Walsh, 1864

##### Ecological interactions

###### Feeds on

Inquiline of: galls of *Andricusquercuslaurinus* Melika & Pujade-Villar, 2009; galls of *Callirhytiscornigera* (Osten-Sacken, 1865); galls of *Callirhytisquercusgemmaria* (Ashmead, 1885); galls of *Callirhytisquercuspunctata* (Bassett, 1863); undetermined galls on *Quercusincana* and other Quercussect.Lobatae spp.

##### Distribution

United States: District of Columbia, Indiana, Michigan, Missouri, New Jersey, New York, Virginia

#### Synergus linnei

Lobato-Vila & Pujade-Villar, 2021

B7B6A4F5-DF55-55F8-BBE4-2289D4938D3B

##### Ecological interactions

###### Feeds on

Inquiline of: small, round galls of *Disholcaspis* sp. on twigs of *Quercus* sp.; small, striped, round, undetermined galls on twigs of *Quercus* sp. (see Lobato-Vila and Pujade-Villar 2021)

##### Distribution

Mexico: Querétaro, Zacatecas

#### Synergus longimalaris

Pujade-Villar & Lobato-Vila, 2017

6080342E-D20B-5466-83E7-869B59DF587E

##### Ecological interactions

###### Feeds on

Inquiline of: galls of *Amphibolipsdampfi* Kinsey, 1937; galls of *Amphibolips* sp. on *Quercusaffinis* Scheidw., *Q.conzattii* Trel., and *Q.glabrescens* Benth.; galls of *Amphibolips* sp. on *Quercus* sp.; galls of *Andricustecturnarum* species group on *Quercus* sp. (see Lobato-Vila and Pujade-Villar 2021)

##### Distribution

Mexico: Hidalgo, Jalisco, México, Michoacán de Ocampo, Morelos

#### Synergus longiscapus

Pujade-Villar & Lobato-Vila, 2017

118E61AA-D9C4-53B2-9111-A0EBA019B519

##### Ecological interactions

###### Feeds on

Inquiline of: galls of *Disholcaspis* sp. on *Quercuscandicans* Née, *Q.eduardi* c.f., *Q.emoryi* Torr., *Q.glabrescens* Benth., *Q.laeta*, and *Q.rugosa*; galls of *Andricus* sp. on *Quercuscandicans* Née, *Q.eduardi* c.f., *Q.emoryi* Torr., *Q.glabrescens* Benth., *Q.laeta*, and *Q.rugosa*; unidentified subellipsoidal galls on the leaves of on *Quercuscandicans* Née, *Q.eduardi* c.f., *Q.emoryi* Torr., *Q.glabrescens* Benth., *Q.laeta*, and *Q.rugosa*; galls of *Neuroteruseugeros* Pujade-Villar; galls of the *Andricusgeorgei* and *Andricusmaesi* groups; galls of *Andricussphaericus* Pujade-Villar; galls of undetermined species of *Andricus*, *Cynips*, and *Disholcaspis* on Quercussect.Quercus spp. (see Lobato-Vila and Pujade-Villar 2021)

##### Distribution

Mexico: Ciudad de México, México, Morelos, Nuevo León, Oaxaca, Tlaxcala, Veracruz de Ignacio de la Llave, Zacatecas

#### Synergus macrackenae

Lobato-Vila & Pujade-Villar, 2021

5EE2D9E0-D07B-5CAA-9D63-286C019F38A5

Synergus nigro-ornatus McCracken & Egbert, 1922

##### Ecological interactions

###### Feeds on

Inquiline of: galls of *Loxaulushyalinus* Pujade-Villar & Melika, 2014; galls of *Andricusfusiformis* Pujade-Villar, 2014; galls of *Neuroterus* sp. on leaves of *Quercusobtusata*

##### Distribution

Mexico: México, Michoacán de Ocampo

#### Synergus medullae

Ashmead, 1885

F7386081-A727-5C21-BACA-BD03A86E7296

##### Ecological interactions

###### Feeds on

Inquiline of: galls of *Zapatellaquercusmedullae* (Ashmead, 1885) on *Quercusincana* Bartram (= *Q.cinerea* Raf.), *Q.marilandica* (L.) Münchn. and *Q.myrtifolia* Willd.

##### Distribution

United States: Florida

#### Synergus mendax

Walsh, 1864

E3C8F2DE-8AC9-5439-9C08-ED3566C5801C

Synerges [sic] *mendax* Walsh, 1864

##### Ecological interactions

###### Feeds on

Inquiline of: galls of *Callirhytisquercuspunctata* (Bassett, 1863)

##### Distribution

United States: Illinois

#### Synergus mexicanus

Gillette, 1896

C6CC0D16-88CE-58FA-9D35-91D057B04153

Synophrus mexicanus (Gillette, 1896) | *Synergusmexicana* Gillette, 1896 | *Synergusdugesi* Ashmead, 1899 | *Synergusmultiplicatus* Fullaway, 1911 | *Saphonerusbrevis* Weld, 1926 | *Synergusbrevis* (Weld, 1926)

##### Ecological interactions

###### Feeds on

Inquiline of: "woody tuberous galls and fusiform twig swellings ... of oaks from both *Quercus* and *Lobatae* sections" (Lobato-Vila and Pujade-Villar 2021); galls of *Andricuschrysolepidicola* (Ashmead, 1896) (= *Andricuskelloggi* (Fullaway, 1911)) on *Quercusdouglasii* Hook. and Arn. (Quercus section) (see Lobato-Vila and Pujade-Villar 2021).

##### Distribution

United States: Arizona, California, New Mexico; Mexico: Ciudad de México, Guanajuato, Hidalgo, México, Zacatecas

#### Synergus nigroornatus

McCracken & Egbert, 1922

EFCAD951-8619-51B0-B27D-49DDEE094F8F

##### Ecological interactions

###### Feeds on

Inquiline of: galls of *Heteroecuspacificus* (Ashmead, 1896)

##### Distribution

United States: California

#### Synergus oaxaquensis

Lobato-Vila & Pujade-Villar, 2021

4558856C-6E8D-5A4C-B873-C72B9950F43B

##### Ecological interactions

###### Feeds on

Inquiline of: galls of an undetermined genus, possibly *Andricus* or *Loxaulus*, on twigs of *Quercusobtusata* (see Lobato-Vila and Pujade-Villar 2021)

##### Distribution

Mexico: Oaxaca

#### Synergus obtusilobae

(Ashmead, 1885)

AE96A876-1598-5F79-82EC-2C026FEFC161

Ceroptres obtusilobae Ashmead, 1885

##### Ecological interactions

###### Feeds on

Inquiline of: undetermined gall on *Quercusstellata*; undetermined gall on *Quercusalba* L. (see Lobato-Vila and Pujade-Villar 2021)

##### Distribution

United States: District of Columbia, Florida, Missouri, Virginia

#### Synergus ochreus

Fullaway, 1911

128EC4E4-F2D3-517F-8889-2E5218B2FFAD

##### Ecological interactions

###### Feeds on

Inquiline of: galls of *Besbicusconspicuus* (Kinsey, 1930) (= Cynips (Besbicus) multipunctata var. conspicua) on *Quercuslobata*; galls of *Amphibolipsquercuspomiformis* (Bassett, 1881); galls of *Disholcaspiseldoradensis* (Beutenmüller, 1909); galls of *Disholcaspis* sp. on *Quercusoblongifolia*; undetermined galls on *Quercuslobata* and *Q.dumosa* (see Lobato-Vila and Pujade-Villar 2021)

##### Distribution

United States: Arizona, California

#### Synergus oneratus

(Harris, 1841)

D3603921-3755-5B07-9D7D-2F4EF28F84BE

Cynips oneratus Harris, 1841 | *Synergusgarryanus* Gillette, 1893 | *Synergusoneratuscoloradensis* (Gillette, 1896) | Synergusoneratusvar.coloradensis Gillette, 1896

##### Ecological interactions

###### Feeds on

Inquiline of: galls of *Andricus* (=*Adleria*) *quercusstrobilana* (Osten-Sacken, 1862); galls of *Atruscabrevipennata* (Gillette, 1893); galls of *Disholcaspisquercusglobulus* (Fitch, 1859); galls of *Disholcaspisrubens* (Gillette, 1893); galls similar to those of *Disholcaspisperniciosa* (Bassett, 1890) (= *Holcaspismonticola* Gillette, 1893) on *Quercusgarryana*; galls of *Disholcaspiseldoradensis* on *Quercuslobata* and *Q.dumosa*; galls of *Disholcaspisperniciosa* on *Quercusgambelii*; galls of *Disholcaspisrubens* on *Quercusxundulata*; galls of *Acraspiserinacei* (Beutenmüller, 1909); galls of *Acraspispezomachoides* Osten Sacken, 1862; galls of *Philonixnigra* Gillette, 1889; galls of *Atruscaquercuscentricola* (Osten Sacken, 1861); galls of *Andricusrobustus* Weld, 1926; galls of *Andricusbiconicus* Weld, 1926; galls of *Disholcaspisquercusmamma* (Walsh, 1869) (see Lobato-Vila and Pujade-Villar (2021) and references therein)

##### Distribution

United States: California, Colorado, District of Columbia, Iowa, Illinois, Kentucky, Massachusetts, Michigan, Missouri, New Jersey, New York, Ohio, Oregon, Tennessee, Washington; Canada: Ontario

#### Synergus pacificus

McCracken & Egbert, 1922

B9C5E632-A05E-5351-84F2-F3B70DFEDE19

Synergus profusus McCracken & Egbert, 1922

##### Ecological interactions

###### Feeds on

Inquiline of: galls of *Heteroecuspacificus* (Ashmead, 1896) (= *Andricuspacificus*) on *Quercuschrysolepis*; galls of *Disholcaspiscanescens* (Bassett, 1890) on *Quercusdouglasii* (see Lobato-Vila and Pujade-Villar 2021 and references therein)

##### Distribution

United States: California

#### Synergus personatus

Lobato-Vila & Pujade-Villar, 2021

6B3DB4F2-AF39-5528-AAC9-AFDAFAB19876

##### Ecological interactions

###### Feeds on

Inquiline of undetermined species of *Atrusca* on *Q.microphylla* and *Q.glabrescens*

##### Distribution

Mexico: México, Tlaxcala

#### Synergus pomiformis

Ashmead, 1885

6584B25F-4B35-5925-8C65-857F491E56C6

Ceroptres pomiformis Ashmead, 1885 | *Synergusflavus* Kieffer, 1904 | *Synergusvaricolor* Fullaway, 1911 | *Synergusvariegatus* McCracken & Egbert, 1922

##### Ecological interactions

###### Feeds on

Inquiline of: galls of *Callirhytisquercuspomiformis* (Bassett, 1881) (= *C.maculipennis* Kieffer, 1904) on *Quercuswislizeni* A. DC. and *Q.agrifolia* (*Lobatae* section); *Amphibolips* sp. on *Quercusagrifolia* (Lobato-Vila and Pujade-Villar 2021)

##### Distribution

United States: California; Mexico: Baja California

#### Synergus pseudofilicornis

Lobato-Vila & Pujade-Villar, 2018

1B828638-BB86-5516-AC24-C33EF965F614

##### Ecological interactions

###### Feeds on

Inquiline of: galls of *Andricusquercuslaurinus* Melika & Pujade-Villar, 2009 (asexual generation) on *Quercusaffinis* and *Quercuslaurina*; galls of *Andricusquercuslaurinus* (sexual generation); other tuberous galls initiated by *Andricus* spp.; woolly galls induced by *Andricus* spp. and *Striatoandricus* spp.; non-woolly galls induced by *Andricus*, *Atrusca*, *Cynips*, *Dros*, *Neuroterus* on *Quercus* spp. (sections *Quercus* and *Lobatae*) (Lobato-Vila and Pujade-Villar 2021)

##### Distribution

Mexico: Ciudad de México, Hidalgo, México, Morelos, Nuevo León, Puebla, Tlaxcala, Veracruz de Ignacio de la Llave

#### Synergus punctatus

Gillette, 1896

E5B42ECB-F50A-5078-A2D1-F3F4770CD9E0

Synergus punctata Gillette, 1896

##### Ecological interactions

###### Feeds on

Inquiline of: galls of *Disholcaspisrubens* (Gillette, 1893) and *Disholcaspiseldoradensis* (Beutenmüller, 1909) and *Biorhizaeburnea* (Bassett, 1890) (= *Dryophantaglabra* Gillette, 1894) on *Quercusarizonica* Sarg., *Q.dumosa* Nutt. (= *Q.durata*), *Q.gambelii* Nutt., *Q.garryana* Dougl. ex Hook., *Q.lobata* Née, *Q.oblongifolia* Torr., *Q.rugosa*, *Q.toumeyi* Sarg., *Q.turbinella* Greene and *Q.undulata* Torr.; galls of *Andricusnigricens* (Gillette, 1888); galls of *Philonixnigra* (Gillette, 1889); galls of *Acraspiserinacei* (Beutenmüller, 1909); galls of *Acraspismacrocarpae* Bassett, 1890; galls of *Andricusrobustus* Weld, 1926 (Lobato-Vila and Pujade-Villar 2021)

##### Distribution

United States: California, Colorado, Illinois, Iowa, Kansas, Kentucky, Missouri

#### Synergus quercuslana

(Fitch, 1859)

4B1A65BB-9314-558C-83EE-EDEB47118087

Cynips quercus-lana Fitch, 1859 | *Andricuslana* Ashmead, 1885 | *Synerguslana* Cresson, 1887

##### Ecological interactions

###### Feeds on

Inquiline of *Andricusquercusflocci* (Walsh, 1864)

##### Distribution

United States: Iowa, New York

#### Synergus reniformis

McCracken & Egbert, 1922

B4EFBBF6-7AFC-5C5D-9EF4-A5DFC79DF0D6

Synergus magnificus Weld, 1957

##### Ecological interactions

###### Feeds on

Inquiline of: galls of Disholcaspis?reniformis (McCracken & Egbert, 1922) on *Quercusvaccinifolia*; galls of Andricus?truckeensis (Ashmead, 1896) on *Quercuschrysolepis*; unknown galls on *Quercuschrysolepis* and *Quercusvaccinifolia* (Lobato-Vila and Pujade-Villar 2021)

##### Distribution

United States: California, Oregon; Mexico: Baja California

#### Synergus ruficephalus

Lobato-Vila & Pujade-Villar, 2021

AEE6DE02-D4E2-5D65-A67E-C0959C80326D

##### Ecological interactions

###### Feeds on

Inquiline of: galls induced by *Andricus*, possibly *A.quercuslaurinus* Melika & Pujade-Villar, 2009, on *Quercuscrassipes* (Lobato-Vila and Pujade-Villar 2021)

##### Distribution

Mexico: México

#### Synergus rutulus

McCracken & Egbert, 1922

B8BA6171-D1ED-5667-A377-4FCE1FC4338B

##### Ecological interactions

###### Feeds on

Inquiline of *Disholcaspisplumbella* Kinsey, 1920

##### Distribution

United States: California

#### Synergus shorthousei

Lobato-Vila & Pujade-Villar, 2019

01B740BE-A163-5D1F-A3CB-BA3C9CBD2859

##### Ecological interactions

###### Feeds on

Inquiline of: "woody tuberous galls of different *Andricus* species on twigs of oaks of both *Quercus* and *Lobatae* sections" (Lobato-Vila and Pujade-Villar 2021)

##### Distribution

Mexico: Guanajuato, Hidalgo, México, Michoacán de Ocampo, Oaxaca, Zacatecas

#### Synergus stelluli

Burnett, 1976

A7A16D67-E928-5141-B77D-165A7AD3FF51

##### Ecological interactions

###### Feeds on

Inquiline of: galls of *Andricusstellulus* Burnett, 1974

##### Distribution

United States: California

#### Synergus striatifrons

Pujade-Villar & Lobato-Vila, 2017

CCACDB43-2B47-562C-9A4E-48C36032BDCB

##### Ecological interactions

###### Feeds on

Inquiline of: galls of *Amphibolipshidalgoensis* Pujade-Villar & Melika, 2011 (asexual generation) on *Quercuseduardi* and *Q.crassipes*; galls of *Amphibolipszacatequensis* Melika & Pujade-Villar, 2011 on *Quercuseduardi* and *Q.crassipes*; galls of *Amphibolipscibriani* Pujade-Villar, 2018 (asexual generation) on *Quercuscrassipes* (see Lobato-Vila and Pujade-Villar 2021)

##### Distribution

Mexico: Ciudad de México, Hidalgo, México, Tlaxcala, Zacatecas

#### Synergus succinipedis

(Ashmead, 1885)

E2DB0B5E-5F07-5B52-8E9C-6409BC07FD70

Ceroptres succinipedis Ashmead, 1885

##### Ecological interactions

###### Feeds on

Inquiline of: galls of *Disholcaspisquercusvirens* (Ashmead, 1881)

##### Distribution

United States: Florida

#### Synergus tenebrosus

Lobato-Vila & Pujade-Villar, 2019

2E89A9BB-D6CC-5C0C-AD33-689D5A8459E5

##### Ecological interactions

###### Feeds on

Inquiline of: woody tuberous twig galls of *Andricus* sp. on *Quercusmexicana* Bonpl. (*Lobatae* Section) and *Q.rugosa* (*Quercus* section)

##### Distribution

Mexico: Guerrero, México

#### Synergus villosus

Gillette, 1891

E027D244-F0EC-5A88-A883-3AA7BD14B72B

##### Ecological interactions

###### Feeds on

Inquiline of: galls of *Acraspisvillosa* Gillette, 1888, *Callirhytislanata* (Gillette, 1891), and *Dryocosmusimbricariae* (Ashmead, 1896)

##### Distribution

United States: Illinois, Iowa

#### Synergus virentis

(Ashmead, 1885)

E7A7CFC1-064D-5579-810E-8E86B2A96B2C

Ceroptres virentis Ashmead, 1885

##### Ecological interactions

###### Feeds on

Inquiline of: galls of *Disholcaspisquercusvirens* (Ashmead, 1881)

##### Distribution

United States: Florida

#### Synergus walshii

Gillette, 1896

4C070962-811E-5100-8ABF-BA45484F4DF0

Synophrus albipes Walsh, 1864 | *Synergusalbipes* (Walsh, 1864)

##### Ecological interactions

###### Feeds on

Inquiline of: galls of *Andricusignotus* (Bassett, 1900), *Andricuspattoni* (Bassett, 1881), *Andricusquercusflocci* (Walsh, 1864), *Phylloterasvolutellae* Ashmead, 1897, and *Phylloteraspoculum* (Osten Sacken, 1862)

##### Distribution

United States: Illinois, Iowa, Kentucky, Michigan, Missouri

#### Synergus weldi

Lobato-Vila & Pujade-Villar, 2021

E967F161-2B81-5650-BBFB-33212FBE0FF4

##### Ecological interactions

###### Feeds on

Inquiline of: galls of *Andricus* sp. on twigs of *Quercusmexicana* (Lobato-Vila and Pujade-Villar 2021)

##### Distribution

Mexico: Guanajuato

## Discussion

Examination of cynipids from the covered geographic range yields 170 valid species amongst 323 published taxonomic names (see Table 1).

Kinsey and Ayres (1922) apparently amended the original spelling of Ashmead's *Diplolepistuberculator* as *Diplolepistuberculatrix* without explanation. The amended name was subsequently carried forward in publications by other authors, particularly in Burks (1979). We treat Kinsey and Ayres's spelling as an unjustified emendation , as described in Article 33.2.3 of the International Code of Zoological Nomeclature (Fourth Edition) and revert to the spelling to Ashmead's original *Diplolepistuberculator*.

Our experience in developing this catalogue reveals the need for a better mechanism to record and share data on extended phenotypes and for fine-grained trait data more generally. A model has been developed for the semantic representation of phenotype data (Balhoff et al. 2013, Mikó et al. 2014), for example, and further development of the Taxon Description extension or even a new phenotype-focused Darwin Core extension might be a convenient vehicle for sharing these data.

## Supplementary Material

40371188-0380-5CBC-918E-037258C8812D 10.3897/BDJ.9.e68558.suppl1 Supplementary material 1 Catalogue of the Cynipidae of North America Data type taxon names, distributions, biotic associations, vernacular names Brief description A catalogue of the Aulacideini, Ceroptresini, Diastrophini, Diplolepidini, Phanacidini, and Synergini (Hymenoptera: Cynipidae) of North America, including information about the taxon name history, vernacular names, biotic associations, and geographic regions in which these species occur. The checklist was created using Darwin Core data standard, including Darwin Core Extensions for Taxon Description, Literature References, Vernacular Names, and Species Distribution. A glossary of gall morphology is also included. The data are compressed into a Darwin Core Archive. File: oo_564492.zip https://binary.pensoft.net/file/564492 Nastasi, L. F. and Deans, A. R.

## Figures and Tables

**Table 1.** Table 1: Species diversity by country

Tribe	Genus	Total Species	United States	Canada	Mexico
** Aulacideini **		21	20	6	0
	* Antistrophus *	10	10	1	0
	* Aulacidea *	10	9	4	0
	* Liposthenes *	1	1	1	0
** Ceroptresini **		19	13	1	7
	* Buffingtonella *	1	1	0	0
	* Ceroptres *	18	12	1	7
** Diastrophini **		25	20	11	1
	* Diastrophus *	14	12	8	0
	* Periclistus *	7	5	2	0
	* Synophromorpha *	4	3	1	1
** Diplolepidini **	* Diplolepis *	34	34	16	0
** Phanacidini **	* Phanacis *	2	2	1	0
** Synergini **		69	44	2	29
	* Saphonecrus *	2	2	0	0
	* Synergus *	67	42	2	29
**Total species**		**170** (Total)	**133** (USA)	**38** (CAN)	**37** (MEX)
